# Comment on Impact of the Percentage of Overlapping Surgery on Patient Outcomes: A Retrospective Cohort Study of 87,000 Surgical Cases

**DOI:** 10.1097/AS9.0000000000000303

**Published:** 2023-06-28

**Authors:** Grant C. Lynde, Eli Mlaver, Jesse A Codner, Jyotirmay Sharma

**Affiliations:** From the † Department of Surgery, Emory University, Atlanta, GA.

We read with great interest the manuscript titled “Impact of the Percentage of Overlapping Surgery on Patient Outcomes: A Retrospective Cohort Study of 87,000 Surgical Cases.”^1^ We want to thank the authors for contributing to our understanding of the significance of continuous attendance by an attending surgeon. While this manuscript provides insights into the significance of case overlap, some key factors are missing from the analysis that may have significant implications for patient outcomes.

We do not know what the overlapping activities were and the degree of complexity, nor do we know the level of training of the individuals performing these activities or the experience/years in practice of the attendings studied. In our era of graduated responsibilities, it would be expected that a senior resident would be able to perform a significant amount of a case without significant input from the attending surgeon. Furthermore, as surgical education transitions to competency-based evaluation, it is increasingly true that the *expectation* of graduating residents is a near-independent management of common general surgical presentations. Aside, we would intuitively hypothesize that attending surgeons with more years’ experience would be more comfortable overseeing overlapping operations, and an exploration of any “inflection point” in this association and of the associations with patient outcomes would be informative. There is also information that we need to know to understand the relevance of this manuscript. For example, we do not know what percentage of any case the attending surgeon was present, even when there was no competing surgical case. The authors did not exclude emergency cases, which would be a significant confounder. The authors do not comment on what percentage of included cases had planned and/or indicated hand-offs to a second surgical specialty that may be led by a secondary attending surgeon. And finally, there was no risk adjustment performed for preexisting medical conditions. It would be expected that a surgeon would not schedule significant overlapping cases when they are concerned about the patient’s condition.

While the data presented are compelling, the story presented is not complete. The observation that overlapping cases *lessened* complications would indicate that important confounders were not addressed in the analysis. With an incomplete analysis that does not take into consideration the aforementioned factors, these premature conclusions can be misconstrued by payors and policymakers, and perhaps impact reimbursement and scope of practice.

## ACKNOWLEDGMENTS

All the authors participated in writing of the letter.
